# ‘You’re disabled, why did you have sex in the first place?’ An intersectional analysis of experiences of disabled women with regard to their sexual and reproductive health and rights in Gujarat State, India

**DOI:** 10.1080/16549716.2017.1290316

**Published:** 2017-05-02

**Authors:** Laura Dean, Rachel Tolhurst, Renu Khanna, Kate Jehan

**Affiliations:** ^a^ Department of International Public Health, Liverpool School of Tropical Medicine, Liverpool, UK; ^b^ SAHAJ Society for Health Alternatives, Vadodara, India; ^c^ Department of Public Health and Policy, University of Liverpool, Liverpool, UK​

**Keywords:** Disability, intersectionality, sexuality, qualitative, gender

## Abstract

​**Background**: Globally, disabled people have significant unmet needs in relation to sexual and reproductive health (SRH). Disabled women in India face multiple discrimination: social exclusion, lack of autonomy with regard to their SRH, vulnerability to violence, and lack of access to SRH care. While they may face shared challenges, an intersectional perspective suggests that considering disabled women as a uniform and ‘vulnerable’ group is likely to mask multiple differences in their lived experiences.

**Objective**: To explore commonality and heterogeneity in the experiences of disabled women in relation to their SRH needs and rights in Gujarat State, India.

**Methods**: We conducted 22 in-depth qualitative interviews with women between the ages of 18 and 49 with any form of self-identified disability. Intersectionality was used as a lens for analysis and in sampling.

**Results**: Findings explore the experiences of disabled women in a number of different spheres related to decision making and SRH service use.

**Conclusions**: Recognising heterogeneity is critical to inform rights-based approaches to promote SRH and rights for all disabled women. This suggests a need to encourage strategic alliances between social movements for gender equity and SRH and disability rights, in which common interests and agendas can be pursued whilst recognising and respecting differences.

## Background

Globally, disabled people have substantial unmet needs in relation to sexual and reproductive health (SRH) [1]. Unmet need is significantly shaped by discourses that construct disabled people, in particular disabled women, as having no sexual desires or agency and therefore as unlikely to have SRH needs [2–5]. Neglect of SRH is a violation of disabled women’s rights [6]. In addition, disabled women often face numerous demand- and supply-side barriers in accessing SRH care, including physical barriers, lack of adaptable equipment, negative treatment from staff, unequal allocation of time and money within the household for their care, and lack of affordability of care through exclusion from employment or income-generating activity [2,7–12]. At the same time, gendered norms in India place an expectation on women to become childbearing wives, but the stigma disabled women face frequently excludes them from performing this social role [13,14]. Disabled women in India may therefore face multiple discrimination: social exclusion, lack of autonomy over their SRH, vulnerability to violence, forced sterilisation and lack of access to SRH care [13,15–17].

While disabled women in India face a number of shared challenges, an intersectional perspective suggests that to consider them as a uniform group who are ‘de-sexualised, feminised and powerless’ [18, p. 1827] is to inaccurately represent their lived experiences, and likely to mask multiple differences. Prioritising the impact of gender alone on disabled sexuality may also be an oversimplification. Rather, gender is one of a number of social inequities (including wealth, class, or caste) that interact to create complex positionalities within a nexus of power relations. These cross-cutting social structures of inequity generate layers of oppression and dominance, as well as spaces for the exercise of agency and resistance that shape disabled women’s expressions of their sexuality and SRH outcomes [18]. In recent decades, this intersectional approach has emerged as increasingly important for interrogating the interaction of social identities with systemic systems of oppression [19]. While health disparities have typically been explored by disaggregating vulnerabilities, prioritising one category over another, or assuming a cumulative effect of multiple vulnerabilities, an intersectional analysis acknowledges the likely complexity of these interactions [19]. It demands a deeper exploration of social processes and structures and how they interrelate at both macro and micro level to shape lived experiences [20–23]. Critiques of intersectional analysis have identified potential risks of losing feminist insight or of presenting individual characteristics as ‘fixed’ ‘tiers’ of vulnerability, rather than fluid and mutually constituting positionalities in social power relations [21,23]. An effective intersectional approach is one that is non-additive, resists essentialising, and is concerned with implications for social action [20–22]. Whilst we aim to move away from the dominance of one social category [22–25], we prioritise gender as an analytical entry point in relation to disabled women’s SRH [18].

This paper explores the experiences of disabled women in relation to their SRH needs and rights in Gujarat, India from an intersectional perspective. By using an intersectional lens, this study explores the diversity as well as commonalities in women’s experiences. We hope that reflections on disabled women’s experience contribute to the evidence base required to develop more nuanced, context-specific interventions whilst moving beyond the homogeneous analytic category of ‘disabled women’.

##  

### Disability in the Indian context

There are over 26 million people living with disability in India, of which approximately 12 million are women [26]. The disability rights movement in India has been growing since 1970 [27], but in mainstream contexts, the physical environment remains largely inaccessible and stigmatisation continues [14,28]. Globally, increasing evidence of the links between poverty and disability suggests that in most low- and middle-income countries (LMICs), people living with disability are poorer than non-disabled people [29,30]. Poverty is linked to exclusion from the workforce, lack of access to education, ongoing social marginalisation, and (direct and indirect) costs of healthcare [29,30]. Many disabled women in India occupy diminished material worlds and rely on informal economic activities for daily survival [31].

Conceptual models of disability can be broadly categorised between the medical and the social [14,29,32]. Medical or rehabilitation models construct disability as ‘defects or impairments that need fixing’ [29]. Social models, by contrast, argue that societal contexts render such impairments disabling [33]. Moral constructions of disability have a long history in India, with impairments understood as ‘retribution’ for previous ‘deceit, mischief and evil’, rendering the impaired person ‘less capable’ [14,34]. Impairment is frequently constructed negatively in India, and there has been a strong preference for rehabilitative or medical interventions to ‘correct’ impairment, with little attention paid to the disabling impact of the social context [14]. By contrast, this study takes a rights-based approach to disability, which values disabled people as equal to the able-bodied and accepts their rights as inalienable [29]. Focusing on how social and economic positions and processes adapt for persons living with disability, this approach also understands disability as socially constructed, in line with the ‘social model’ of disability [35].

### Sexuality, disability, and gender in India

Historically in India, as in other contexts across the globe, the public representation and discussion of sexuality have oscillated between periods of greater or lesser openness and periods of prohibition and taboo. For disabled people, communication about sexuality and SRH and rights is likely to have been especially silenced in conservative eras [36–38]. Recent decades have nevertheless seen SRH increasingly included within policy and programming [39]. Despite this, significant challenges to women claiming SRH rights remain socially shaped by interactions between gender, poverty, religious prohibition, and social norms and values [39,40]. In particular, studies in Gujarat identify poor access to SRH services being linked to inequities produced by multiple forms of disenfranchisement, including: a reduction in women’s financial autonomy and decision-making to seek care; lack of identified or perceived SRH needs; stigma and social taboos; and limited household resources [40,41].

Globally, the few qualitative studies exploring the SRH needs and rights of disabled people identify a gap between SRH needs and ability to access SRH services [2].^1^ ^1^SRH services include but are not limited to: maternal and child health services; family planning services; S.T.I screening; and gynaecology services.​ Limited access derives from similar social, infrastructural, and economic barriers that ‘non-disabled’ women typically experience. However, some studies argue that these issues are compounded when they intersect with negative stereotypes of disabled women’s sexuality [2–5,8–12,42]. A study in north India with disabled women identified similar barriers in that context, though differences within the group of disabled women were underexplored [8]. The phenomenon of ‘desexualised subjectivity’ [43] has been linked by Indian social analysts to the long-established ‘moral’ construction of disability, in which impairment is perceived as retribution for the past and disabled bodies as dysfunctional [14,28,44,45]. A study conducted in Delhi with disabled adolescents emphasised the great variety in young people’s ability to express their sexuality in oppressive environments, highlighting that some disabled youth are able to challenge their ‘desexualised subjectivity’ by expressing their sexual needs and desires [43,44]. The limited qualitative evidence base suggests that deeper understandings of the enabling factors or environments for such challenges to dominant discourses on disability and sexuality are needed.

Critically, as discourses around gender and sexuality gain momentum in India, it is important that mainstream activism and service provision are inclusive of disabled women’s experiences, needs, and priorities. Vaidya [36] describes the goal of disabled women’s sexual and reproductive rights as one that should transcend the right to decide about childbirth to include recognition of themselves as sexual beings. Furthermore, claiming the social position of motherhood (in a context where motherhood is highly valorised) allows a transition from dependency to independence; the role of motherhood can provide space for disabled women to resist dominant discourses surrounding disability and sexuality [42]. Nevertheless, both felt and enacted stigma surrounding disability and motherhood remain [36,42,46]. Neither is it helpful to uphold motherhood as a form of legitimisation, where women have historically struggled to seek legitimacy beyond this role (and for many women acceptance through motherhood may not be an option).

## Methods

### Study design, study sites, and sample

There are limited examples of the practical application of an intersectional perspective in LMICs. Existing studies tend to apply intersectionality as an analytical lens [20,21]. The few studies that apply intersectionality principles to wider study design focus on the identification of social categories as an entry point for sampling and analysis, whilst simultaneously prioritising heterogeneity across multiple axes within and across such categories [20,21]. In line with this approach, within our study sample heterogeneity was prioritised across the ‘entry point’ social categories of gender and disability. Maximum variation was sought among participants, including a range of socio-economic backgrounds and impairments [47]. Narrative and less structured methods are also cited as critical approaches in intersectional and disability research as they allow participants to construct their own story [21,48]. We therefore used an interpretivist approach through unstructured in-depth interviews to explore how disabled women’s positionalities shape and are shaped by their experiences [22,49,50]. Study locations were urban Vadodara and Ahmedabad, Gujarat, India.

Participants were sampled purposively against the following selection criteria: women aged 18–49; living with any form of self-identified disability; and living in Vadodara and Ahmedabad, Gujarat, India within the catchment areas of two local non-governmental organisations (NGOs) supporting marginalised populations, including disabled people. Impairment was used to identify participants’ physical condition. However, in line with social approaches to disability, self-identification of disability linked to impairment was imperative for inclusion in the study [51]. The minimum age (18) was chosen because this is the legal age of marriage in India, although unmarried women were included in the study. The maximum age (49) was chosen as the upper end of the standard age range for women of reproductive age [52].

Socio-economic status (SES) was gauged according to participants’ living conditions: women living in urban slums are described as lower SES, while women living outside of urban slums in permanent structures are described as higher SES. Caste was not used as a selection criterion as the partner NGOs who work with diverse vulnerable groups believe that attention to caste reinforces the practice of casteism and promotes caste-based identity politics. Women of lower SES were identified from a sampling frame of all disabled women currently living in urban slums in which the Vadodara-based NGO is working [53]. To identify women of higher SES, purposive sampling through networks of the Vadodara-based NGO was used with further snowball sampling [53]. To increase the sample size of women of higher SES, a sampling frame of women of higher SES that fell within the catchment area of the organisation based in Ahmedabad was obtained from organisation records.

### Data collection and ethics

Data were stored securely throughout. Ethical approval was obtained from the Liverpool School of Tropical Medicine. Due to the vulnerability of participants, emphasis was placed on safeguarding through links with local organisations. Informed consent was sought from participants following an explanation of the research study, clarification that participants were free to withdraw from the study at any time, and assurance that participation was voluntary and anonymous. In one case where a participant was unable to provide consent directly, due to learning disability, consent was obtained from their guardian and assent obtained from the participant prior to inclusion in the study as per recommended practice in disability research [54,55]. Interviews covered a range of topics including: understanding of SRH, experience of using SRH services, and personal relationships. If these topics raised an emotional response for participants the interview was paused. Participants were then asked if they wished to proceed. Participants were also given the option to be linked to relevant support services through the partner NGOs. LD conducted interviews in Gujarati or Hindi, with concurrent translation by one of four translators who had experience working with marginalised populations and had been trained in qualitative methods. Interviews were conducted in participants’ homes by LD and a translator. Interviews lasted approximately one hour.

### Data analysis

Interview recordings were transcribed into Gujarati or Hindi and then translated into English. A selection of transcripts were back-translated to check for accuracy. Data were analysed using thematic analysis with an intersectional lens in that attention was paid to how individual positionalities in terms of SES, marital status, life course, and type of disability shaped and were shaped by experiences of SRH and rights. The diverse sample obtained allowed for interrogation into how axes of power and privilege intersected in different spaces and places to shape experience. Fluidity and collaboration in analysis supported our non-additive approach to intersectionality that allowed researcher reflections on their own positionality within the process. Transcripts were analysed using an inductive framework approach focusing on two main principles: data management (coding and sorting of data) and explanation of data, during which links between themes and codes are explored and situated within wider discourse [56]. The data management phases were completed by LD and the translators, with further interpretation of the data completed by all authors.

## Results

Twenty-two interviews were completed; variation within the sample is highlighted in Table 1. Two thirds of participants were of lower SES. Most participants were married and were mobility disabled.

**Table 1.** Study participant characteristics.

		Mobility disability		Visual disability		Deaf		Learning disability		
	Age	M	UM	M	UM	M	UM	M	UM	Total
Lower SES	18–25	4	2							6
	26–33			1					1	2
	34–41	3	1				1			5
	42–49	1								1
Higher SES	18–25									0
	26–33		1							1
	34–41			2						2
	42–49	4	1							5
Total		12	5	3			1		1	22

Notes: M = Married; UM = Unmarried.

### Whether and whom to marry: autonomy and criteria in decision-making

The majority of women described marriage decision-making as constrained and largely controlled by their parents or guardians. This process was sometimes described favourably, with some participants believing, ‘If parents choose, it’s better’ (Geeta, 18, unmarried, low SES, mobility disabled). Other women did hold divergent views from their guardians, but few were able to act autonomously or influence decision-making. Some women described struggling to obtain permission to marry, whilst others were married against their will. Where disagreements occurred, the bargaining power of respondents appeared in part to be shaped by degree or type of disability, socio-economic background, or a mutually constitutive interaction of the two. Rekha, who has a learning disability, described feeling resigned to her family’s decision that she would not marry:

Not now, they said, no; my papa said no because the sisters are away [married] […] My sister told me not to get married – stay home. (Rekha, 32, unmarried, low SES, learning disabled)

By contrast, Trupti had challenged her parents’ decision to prevent her from marrying and successfully made efforts to find her own partner. Trupti was mobility disabled and of a higher SES than Rekha. Participants of a lower SES who had been granted more autonomy reported a more difficult process in achieving this. Neeta, 40 years old, married, and mobility disabled, reported resorting to a threat to ‘put an end to my life’ if she could not marry the partner of her choice. Some participants perceived that if they were to marry, marriage to another disabled person could be protective. Thus norms of disability, gender, and SES intersect to shape marriage decisions:

If a disabled person gets married to a ‘normal’ person then they may accept or not accept her, or they may also desert her. (Ghaada, 35, married, low SES, mobility disabled)

There is obvious discrimination; if she steps out with her husband who is ‘normal’, she gets stared at, because it is OK for a disabled man to marry a normal woman, but not vice versa. If a normal man is with a disabled woman, people will talk; what is wrong with him? (Trupti, 49, married, high SES, mobility disabled)

### Decision-making in the use of SRH services

Women’s use of SRH services was shaped by both awareness and perceived need. The majority of women reported being aware of SRH services. However, none of those women who were aware of SRH services perceived preventative services to be necessary; SRH services were sought only once problems arose. Most women also perceived SRH services as necessary only after marriage and in the pre- and post-partum period. Women rarely reported making the decision to use SRH services alone, with levels of autonomy apparently shaped by interactions between socio-economic and marital status. The majority of married women of lower SES reported making decisions only after consultation with their spouses, parents, or elders. Unmarried participants of lower SES reported decisions being made without their consultation. Participants of higher SES reported taking decisions on their own, regardless of marital status:

I didn’t talk to anyone. I contacted the doctor directly […] I rung her up and told her that I am coming to meet you. So, it is like that. (Kairivi, 47, married, high SES, mobility disabled)

The majority of participants across socio-economic backgrounds showed a preference for private over government facilities, citing a fear of poor treatment in state-run facilities. Poor treatment was perceived as including ‘impolite’ tones and ‘very insulting’ language, which may or may not link to disability (Susheela, 49, unmarried, high SES, mobility disabled). Participants also associated payment for treatment with improved quality of care:

They charge you more but the treatment is good […] We go for better treatment, so [that] we don’t have any problems. We give more fees, but at least we get well. (Bhavna, 18, unmarried, low SES, mobility disabled)

Those who reported using a government facility were generally of lower SES and attributed this use to a lack of money to pay for private care. One participant explicitly related the opportunity to access good-quality care with economic status:

The one who has money can go to a good doctor who has the information [i.e. skills and services]. The one who doesn’t have money can get their treatment done at the government. (Leela, 24, married, low SES, mobility disabled)

Several women of both high and low SES placed value on accessing a medical practitioner, either governmental or private, who was perceived to ‘understand disabled persons’ (Kairivi, 47, married, high SES, mobility disabled), or who could facilitate specific needs, such as communication through sign language. Some women identified family support as a necessity in accessing services. Smita, who was deaf, was scared to leave the house alone due to fear of being attacked, and reported relying on her sister to accompany her:

[She] is comfortable with only one doctor […] She herself tells the doctor what she is suffering from through sign language. He understands her. [Participant’s sister is translating from sign language on her behalf] (Smita, 34, unmarried, low SES, deaf)

### Experiences of using SRH services

Participants described a range of treatment by staff. Sometimes women attributed either positive or negative treatment to their disability. When treatment was positive, one woman perceived that she received ‘special care once he [the doctor] knew she was disabled’ (Ghaada, 35, married, low SES, mobility disabled, referring to a private facility). Renu discussed how interaction in the clinical encounter was adjusted to her disability: ‘Because I can’t see, they hold my hand and give me treatment in a very proper way’ (39, married, high SES, visually disabled, referring to a private facility). Only women of a higher SES attributed negative behaviour to their disability. Behaviour described in this case tended to refer to the derogatory language medical staff had used, for example:

When my friend underwent delivery, she was yelling in frustration and the nurse was yelling at her, ‘You’re disabled, why did you have sex in the first place?’ (Meena, 32, unmarried, high SES, mobility disabled, referring to a government facility)

Though private facilities were often described more favourably, this was not always the case, and Kamilla’s poor treatment in a private facility evoked such feelings of mistrust that she could not undergo childbirth again:​

That time the nurses used such vulgar words, that those words don’t come to our lips. […] [I] can’t take a risk for the second time. (Kamilla, 43, married, high SES, mobility disabled)

Ghaada reported what she perceived as an unfair withholding of an intrauterine contraceptive device, leaving her without a contraceptive method:

[The doctor] said that the copper T will get trapped inside because of the disability, and it wasn’t inserted. (Ghaada, 35, married, low SES, mobility disabled)

Leela reported a doctor strongly steering her towards a hysterectomy after her second child, on the basis that she was ‘weak’ as a result of her disability. She did not object to this advice, which she felt had supported her own wishes, which had previously been opposed by her husband, thus resisting patriarchal control of her body:

After the first child, I was feeling that I wanted to get operated because there were difficulties […] [But] my husband said why only one child, we should have two at least. (Leela, 24, married, low SES, mobility disabled)

Amongst participants of lower SES, there was a common perception that the doctor’s opinion was always correct and unchallengeable. Conversely, participants of a higher SES were more selective in their interpretation of medical advice:

To a certain percent, the doctor is also right that perhaps she will have a lot of difficulties [due to her disability] and to a certain percent, we think that the [disabled] lady is also right. Both are 50%–50% right. (Susheela, 49, unmarried, high SES, mobility disabled)

Thus disability intersects with SES and gender norms in a paternalistic health system to shape the negotiation of sexual and reproductive rights.

Participants’ experiences of physically accessing SRH services varied. The majority of mobility-disabled participants had attended facilities which were on the ground floor, but others faced difficulties whilst climbing stairs, since often there was no lift available. Physical accessibility challenges were similar regardless of participants’ SES:

I had problems walking. […] I used to climb two storeys as the ward was on the second floor. I had problems climbing the stairs, I thought, this is my problem, so I used to climb the stairs carefully and slowly. (Ghaada, 35, married, low SES, mobility disabled)

Some mobility-disabled participants also reported having to climb onto beds that did not lower, or being carried in a sheet to move between areas as there was no stretcher or wheelchair available:

I was climbing on the high bed and [the doctor] kept asking me to be careful and I told her that I will climb. [The doctor] asked me if I needed support from her, but I said I will climb by myself. (Meena, 32, unmarried, high SES, mobility disabled)

Meena’s determination to climb onto the bed herself and Ghaada’s lack of disclosure to the doctor regarding the accessibility difficulties she faced highlight a perception widespread amongst the women with mobility disability, predominantly those of low SES: their mobility problems were their own, and they needed to develop ways to deal with them.

### Experiences of familial and intimate partner violence

Two participants reported challenges to attaining SRH and rights in the form of domestic physical, emotional, and verbal abuse. Both participants were of a lower SES and had had little access to education. These participants attributed poor treatment to their disability, which rendered them feeling powerless. Anjali’s story demonstrates how her lack of autonomy in her natal family, and violent and controlling behaviour by her husband, interacted and reinforced each other, limiting her access to both mobility and medical care and creating feelings of isolation, vulnerability, despair, and suicidal ideation.

Anjali is mobility disabled and moves around her home by dragging herself. She was married despite ‘telling them [her parents] I will get better offers [if she waited] as my age was hardly 13–14 years’. Her parents felt that, ‘“your legs are like this, you won’t find anyone else” … and didn’t pay any need to my requests’. As a result, she said, ‘my soul [was] forced to get married’. Once married, Anjali sought access to treatment for fertility problems with the permission of her husband. At a later stage, however, her husband ‘stopped the treatment’ and when she asked him why, ‘he won’t give a proper answer. […] Now he beats me […] and has dismantled my tricycle and told me that I should not go here nor there.’ As a result, Anjali has been left to feel ‘I should consume medicines and go off to sleep. Then, at times, I feel like burning myself to die…’ (24, married, low SES, mobility disabled).

## Discussion

Our results highlight that disabled women’s recognition of their rights and ability to control their own sexuality are unevenly constrained due to intersectional gendered power relations. Recognition of needs and rights was key to demanding SRH services. All participants encountered assumptions about their sexuality and reproductive capacities by both family members and professionals. These assumptions were based on notions of their body as impaired, simultaneously drawing on and reinforcing discourses that construct disabled women as non-sexual and, therefore, unable to fulfil the gendered role of a wife and mother [2,8,13,14,28,44]. Although some of our participants challenged these discourses, their opportunities to effectively contest them in their own lives were shaped by intersecting power dynamics including: gender, type and severity of impairment, marital status, and SES [18]. In common with findings from studies with ‘non-disabled’ women [41], disabled women of higher SES in this study described more opportunities to exercise autonomy, challenge constructions of ‘asexuality’, and influence or direct decision-making. This was likely supported by their greater access to both material and social resources, though those decisions were in turn shaped by disability norms and what was considered appropriate. Gender structures and socio-economic conditions intersected with disability to shrink the space for disabled women at both ends of the economic spectrum. To some degree, the disabled ‘role’ which all the women occupied diminished their scope to articulate their sexuality and health needs, or claim their SRH rights. Strategies to challenge restrictive discourses and raise awareness of SRH rights and services need to take into account the diverse ways in which disabled women may require specific practical and strategic support [36]. This support is critical in creating environments in which women might be better able to exercise their agency and to recognise and claim their rights.

Many of the women in our study faced both physical and financial barriers to accessing acceptable services, which varied with SES and social autonomy. Financial barriers to accessing quality healthcare are commonly experienced by lower-SES women in Gujarat [41]. Our intersectional perspective thus highlights how limited provision of physically accessible SRH services interacts with socio-economic inequities in access to quality healthcare in urban Gujarat [57]. Financial inequities may be exacerbated for some disabled women due to unequal distribution of household resources and their relatively low perceived contribution to household finances [9,31,58,59]. Simultaneous action is needed to improve physical and financial accessibility of health services, including SRH services. Despite continued lobbying [59], accessibility for disabled people in public and private buildings remains limited in India, often due to lack of funding and prioritisation [8,60,61]. Advocates have identified the importance of securing funding allocation to improve the physical environment, and have pointed to the potential of combined disability and gender budgeting to achieve this [62]. Increased demand by disabled women may be a necessary driver of efforts to increase the prioritisation of improving physical access.

Prejudice and paternalism in service provision were frequently reported by our study participants, though intersections with expressions of disabled sexuality meant that women perceived this in a range of ways. Although negative staff attitudes are commonly reported, particularly in the public sector in India [41], the experiences of some participants, including treatment denial, emphasised how societal perceptions of disabled sexuality can be expressed in SRH service delivery. Such manifestations often resulted in the denial of sexual and reproductive rights for disabled women, including the right to respectful care and the right to control fertility [6]. However, our findings also highlight that disability is not always ‘additive’ to gender inequity in that sometimes, presumptions around sexuality and SRH needs and rights created a space for women to exercise agency. In existing literature focusing on disabled sexuality, disabled women often emerge as ‘victims’ who are denied SRH rights [2]. This emphasises the benefit of underpinning an intersectional analysis with an interpretivist epistemological approach, enabling us to elicit and prioritise participants’ subjective understanding of their own realities [23]. Encouragement to have a hysterectomy may be perceived as a denial of reproductive rights; however, in Leela’s case this offered an opportunity to pursue her own felt needs and resist patriarchal control over her body. Similarly, some participants expressed their experiences of neither marrying nor having children as fulfilment of their own desires. Whilst this may be interpreted as a possible internalisation of ‘desexualised subjectivity’ [43] and negative societal perceptions of disabled motherhood [36], it may also reflect an opportunity offered by impairment to circumvent restrictive gender roles. In addition, whilst literature has mainly reflected disabled women’s negative experiences of accessing SRH services [2,7,17], this study has identified some women’s positive experiences of interaction with staff and the receipt of ‘special care’ related to their disability. This suggests hope for the creation of more enabling environments. ‘Special care’ was, however, most commonly observed in private facilities. Financial barriers need to be addressed if equity in access to SRH services is to be achieved.

Charlton [63] describes ‘a transformation of consciousness into active resistance’ (p. 11) which is taking place within disability movements globally, and is reflected by rights-based models of disability [64]. Our findings support the importance of such a transformation, noting that such consciousness-building is likely to develop unevenly due to the multifaceted and fluid identities of disabled women. Ghai [31] argues that promotion of strategies that have proved beneficial in the West may be ineffective, ignoring the complex positionalities of disabled women and allowing little space for the development of a post-colonial activism that responds to the Indian context of disability [31,65]. As is commonly described in the discourse around motherhood and disability [36], the use of ‘symbolic capital’ was common in this study to create space to exercise agency [66]: one woman threatened suicide in the pursuit of her right to choose her husband, while another used a doctor’s prejudice to her own advantage (a desired hysterectomy). Critical to strategy development and priority setting are disabled women’s own participation and leadership. This would support transformative efforts to enable disabled women to claim their SRH rights, strengthen their agency, and increase room for manoeuvre in ways that are sensitive to difference and varying strategic interests [67]. Some advocates have argued for the need to build better linkages between women’s rights (including SRH) movements, which are strong in India, and disability rights movements, which are gaining momentum [8]. Alliances may draw productively on ‘transversal’ political approaches to identify shared values and interests, and strategic opportunities to organise and pursue these [68]. These approaches would, importantly, recognise and respect difference, creating space for the pursuit of alternative priorities.

### Strengths and limitations

Study participants were mainly those who were visually disabled, mobility disabled, or deaf. Those with learning disabilities are under-represented, as the majority of women living with learning disabilities were unable to provide assent. Some communication difficulties may have limited the depth of interviews with women who were deaf or who had a learning disability. All participants were identified through local NGOs and therefore women who are not currently engaged with these NGOs or their networks may have been missed. The main strength of this research is the inclusion of a diverse group of disabled women’s experiences.

## Conclusion

This study has used an intersectional perspective to explore heterogeneity in disabled women’s experiences of their opportunities to claim their rights to a sexual life, control of their fertility, and appropriate, respectful SRH care. Recognising heterogeneity is critical to inform rights-based approaches to promote SRH and rights for all disabled women, including advocacy and service provision that are tailored to reflect their varying strategic interests. This suggests a need to encourage strategic alliances between communities working on issues of gender, SRH, and disability, to pursue common interests and agendas of disabled women, whilst recognising and respecting differences. Consciousness-building and continued lobbying for increased resource allocation are critical to challenge restrictive and oppressive discourses, and to ensure that physical environments are adapted to improve access to SRH services.

## Paper context

Disabled women in India are thought to face multiple discrimination in relation to their sexual and reproductive health (SRH). Taking an intersectional approach, we explored the commonality and heterogeneity in the experiences of disabled women in relation to their SRH needs and rights in Gujarat State. Recognising heterogeneity is critical to inform rights-based approaches to promote SRH and rights for all disabled women, including advocacy and service provision that are tailored to reflect varying strategic interests.​
